# Analysing the Impact on Health and Environment from Biogas Production Process and Biomass Combustion: A Scoping Review

**DOI:** 10.3390/ijerph20075305

**Published:** 2023-03-29

**Authors:** Marco Tamburini, Roberta Pernetti, Manuela Anelli, Enrico Oddone, Anna Morandi, Adam Osuchowski, Simona Villani, Cristina Montomoli, Maria Cristina Monti

**Affiliations:** 1 Unit of Biostatistics and Clinical Epidemiology, Department of Public Health, Experimental and Forensic Medicine, University of Pavia, 27100 Pavia, Italy; 2 Unit of Occupational Medicine, Department of Public Health, Experimental and Forensic Medicine, University of Pavia, 27100 Pavia, Italy; 3 Hospital Occupational Medicine Unit (UOOML), ICS Maugeri IRCCS, 27100 Pavia, Italy

**Keywords:** biogas plant, biomass, anaerobic digestate, occupational exposure, health risk

## Abstract

The increasing demand for renewable energy production entails the development of novel green technologies, among them the use of biomass for energy generation. Industrial processes raise new issues regarding emerging risks for the health of people working in biogas plants and of nearby communities. The potential epidemiological and environmental impacts on human health related to biogas plants were assessed by means of a review of the available literature. Nineteen papers published between 2000 and 2022 were identified through electronic database search using search strings. The selected works are epidemiological studies and environmental monitoring studies, which aimed at investigating what are the health risk factors for biogas plant workers and for people living in the surrounding communities. The results of the epidemiological studies revealed a potential exposure to endotoxins and fungi that are associated with respiratory symptoms. Furthermore, the results from the environmental monitoring studies showed significant concentrations of particulate matter, microbial agents, endotoxins, and VOCs in occupational settings. In conclusion, the results of this literature review suggest that further analyses through an integrated approach combining environmental and health data are necessary for a comprehensive understanding of the potential risks associated with the uptake of biogas technology.

## 1. Introduction

The European Commission aims to reach the target of net-zero greenhouse gas emission by 2050 [1]. To comply with this objective, there is a need for significant energy transition based on both the improvement of energy efficiency and the uptake of renewable energy [1]. The significant need for increasing energy production from renewable sources, leads to the development of innovative “green” technologies. Biogas, is recognised by the European Commission as one of the key solutions for both existing buildings and several industrial applications [1]. Biogas production is based on anaerobic digestion (induced by specific mixture of bacteria and additives) of a biomass feedstock. The employed feedstock generally belongs to one of these categories: agricultural products or residues, wooden by-products, manure from livestock rearing, by-products of wastewater treatment, and urban organic waste [2]. In particular, the employed feedstock represents the main factor affecting the level of sustainability of biogas production in terms of both greenhouse gas (GHG) emissions [3] and life cycle assessment (LCA) of plants [4,5,6].

Several studies analysed biogas production technology in terms of costs and benefits, as well as potential environmental impact of biogas. These assessments highlighted a significant reduction in terms of carbon emissions associated with the production and application of biogas energy compared to fossil fuels, but the production may entail the release of potential pollutants that should be further evaluated and studied [7,8,9]. In this regard, the characterization of pollutant emissions, as well as the analysis of potential health implications at different levels (i.e., for workers and nearby communities), represent strategic outcomes to support the increase in social acceptance of this technology across Europe [5,6].

**Figure 1:**
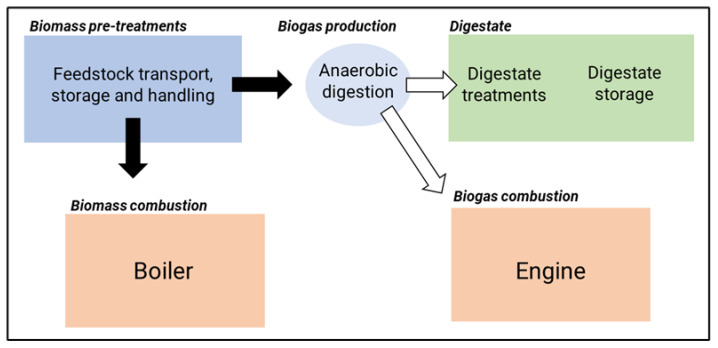
Overview of the main process and monitored activities within the included studies (modified from [10]).

To study the main features of the process, it is possible to break down the operations into three macro-phases that deal with biogas production and biomass handling processing of digestate and biogas or biomass combustion for energy production (Figure 1).

The development of novel technologies and industrial processes presents new technical and organizational variables that can generate traditional occupational risks, which are usually managed through proven standards and existing requirements, as well as new emerging risks, which should be further assessed and deserve targeted analysis [11]. In a biogas plant, in addition to the traditional risks connected with manual handling of loads and, in particular, when handling feedstock and the produced digestate, there may be an exposure to both biological and chemical pollutants during the different phases of the process [12,13,14]. The novelty of the technologies, the limited amount of research available on the topic, the high number of processable feedstocks, the specific design of a facility, and the variety of implemented processes have led to a general difficulty in providing a clear statement on the potential exposure at occupational and residential levels. Therefore, novel technologies need a detailed assessment for the identification and quantification of potential risks to promote the implementation of safe working procedures and to identify variables that need to be controlled to limit the potential threats to workers as well as to the surrounding communities.

This paper aims to identify the potential effects of exposure to chemical and biological pollutants on workers and nearby communities of a biogas plant according to the processed feedstock and technology implemented. The complexity of this process entails several uncertainties that may have a significant impact when modelling the impact of a biogas plant [15]. Therefore, the added value provided by this review relies on the presentation of potential exposure through quantitative data from real biogas plants. In fact, one of the main requirements for the inclusion in the list of selected papers was the availability of data either from on-field monitoring or from an epidemiological study, aiming to collect benchmarks and reference values and to provide a characterization of potential emissions and related effects from existing plants at both occupational and community levels. This is crucial to derive clear statements on the potential risks associated with biogas production plants at both occupational and community levels and, on the other hand, to identify potential benchmarks for the assessment of further sites.

The results reported here provide an initial assessment of the main hazards to be further evaluated on the field.

## 2. Materials and Methods

The analysis was based on the workflow of an electronic database search and followed the Preferred Reporting Items for Systematic Reviews and Meta-Analyses (PRISMA) guidelines [16].

### 2.1. Inclusion/Exclusion Criteria

The research question used for the review was “What are the risk factors for the health of the people working in biogas plants and of the surrounding communities?” Starting from the research question, the inclusion criteria were defined. The framework of this review was based on an analysis of studies that present actual data from on-field environmental monitoring—dealing with biological and chemical pollution due to particulates, gases and volatile organic compounds (VOCs) released by the processes—and epidemiological investigation on populations that are potentially exposed. Moreover, given the limited amount of research currently available on biogas plants, the authors included studies regarding other types of bioenergy technology (namely biomass combustion plants and biorefineries) that also involve the use of biomass feedstock employed in biogas production. 

In order to organize the included studies and provide a homogeneous description, the following classification criteria were adopted:

- Epidemiological studies: studies carried out on a general or specific population (both workers and surrounding communities) to assess the outcome frequency and its potential association with exposure;
- Environmental monitoring studies: studies with pollutant measurements carried out in the workplace or in the surroundings of biogas production sites. The pollutants include biological components, VOCs, and gases and emissions from biogas plants as analysed in Life Cycle Assessment (LCA) analyses;
- Primary studies written in English and published between 2000 and 2022.
- Furthermore, the following exclusion criteria were applied:
- Lack of quantitative data from modelling, monitoring, or epidemiological evaluations available;
- Papers focused solely on pollutant emissions from plants producing energy through biomass combustion or other sources;
- Works presenting analyses on domestic plants, as the focus of this research was on the industrial setting.

### 2.2. Information Sources and Search Strategy

The literature search was carried out by means of diverse electronic sources, i.e., PubMed, Web of Science (WoS), and ScienceDirect (SD). The search strategy was carefully designed to retrieve the most relevant results. Due to the specificity of the three databases employed, for each one, a different search string was built:

PubMed: (health OR human) AND (biomass OR biogas OR anaerobic digestate OR biofuel) AND (hazard OR risk OR exposure OR impact) AND (worker OR resident OR community OR population). Number of results: 1403.

- Web of Science: (ALL = (health)) OR ALL = (human) AND (((ALL = (biomass)) OR ALL = (biogas)) OR ALL = (anaerobic digestate)) OR ALL = (biofuel) AND (((ALL = (hazard)) OR ALL = (risk)) OR ALL = (exposure)) AND ALL = (impact) AND (((ALL = (worker)) OR ALL = (resident)) OR ALL = (community)) OR ALL = (population). Number of results: 845.
- ScienceDirect: (health) AND (biomass OR biogas OR anaerobic digestate) AND (risk OR impact) AND (worker OR community OR population). Number of results: 722.

Primary studies published in English between 2000 and 2022 were eligible for inclusion, and no regional restrictions were applied. Table 1 summarizes the key aspects considered in this review, namely focus, technology, topic, and exposed subjects, and the keywords adopted for the literature search.

The electronic database search was conducted in March 2022. From the screening of eligible studies, the reasons for the exclusion of studies were also reported.

Studies were also identified through citation searching. These articles were regarded as relevant even though they were not identified through the search strings.

### 2.3. Selection Process

Titles, abstracts, and full texts were screened by the research team, i.e., each member of the team screened a part of the whole set of studies. In case of doubt about the selection of a study, a collective decision on the selection was taken.

### 2.4. Data Extraction

For each category, a standardized data extraction sheet using Microsoft Excel 2016 was prepared, where the main information of the studies was collected (e.g., first author’s name, study title, publication year, DOI, country, biomass type, methodology, population involved, exposure, outcome and results, and additional notes).

## 3. Results

### 3.1. Study Selection

**Figure 2:**
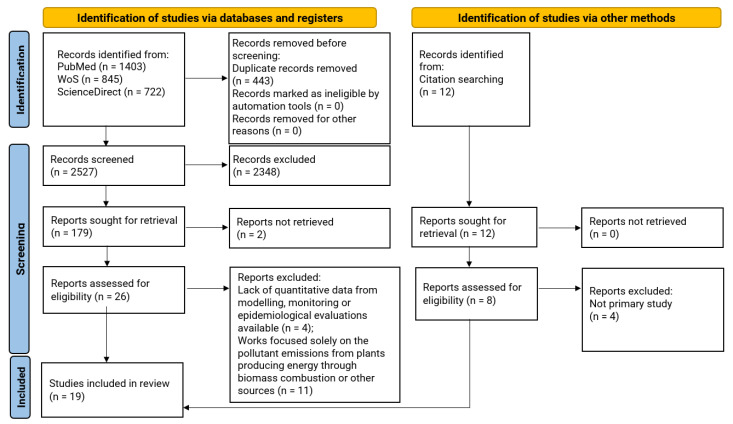
Study selection process. For more information on this flowchart, see [16].

Through the comprehensive literature search, 19 studies were included in this literature review. Eleven of them were identified via the database searches, and eight were identified via citation searching (Figure 2).

The number of the search records retrieved from the three database searches was 2970; additionally, 12 records were identified through cited reference searching. The number of the screened titles/abstracts after duplicate removal was 2527. One hundred seventy-nine full-text papers were analysed, among which 26 were assessed for eligibility. The rest of the papers do not report analyses dealing with potential occupational exposure or impact on the surrounding communities of biogas production plants; thus, they were excluded. Finally, 19 papers were included in the review, as indicated in Figure 2. 

### 3.2. Study Characteristics

**Figure 3:**
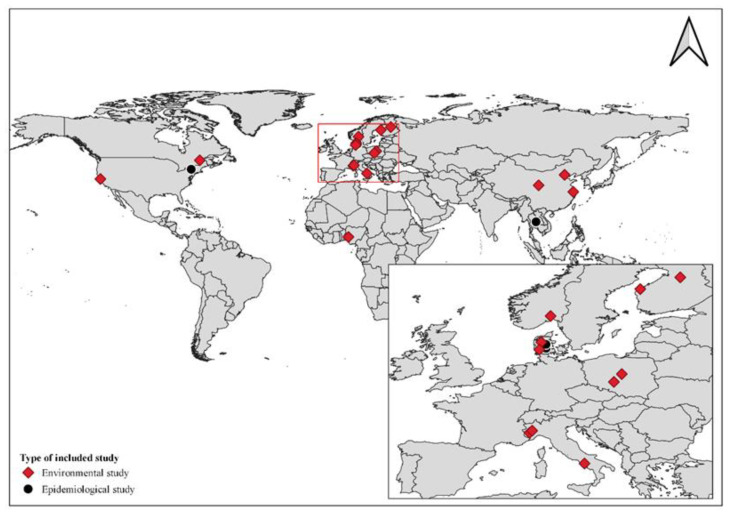
Geographical distribution of the selected studies around the world and in the European countries. Red diamonds: environmental studies; black dots: epidemiological studies.

As shown in Figure 3, the analysed studies were conducted in different geographical contexts: most of them were carried out in Europe (n = 12), Asia (n = 4), and North America (n = 3). Each geographic context shows specific features in terms of level of technical development, availability of feedstock, and technical standards for plant construction and operation; these peculiarities were considered in the analysis of the results. Among the included works, 4 studies are classified as epidemiological studies and 15 as environmental monitoring studies.

### 3.3. Epidemiological Studies

On the basis of the results of the literature search, the health impact of industrial biogas facilities seems to be a scarcely investigated topic from an epidemiological viewpoint. In particular, among the four epidemiological studies included in this section, none of them properly deals with plants where biogas is the sole final product. However, each study addresses certain aspects that may apply to the biogas production process. More specifically, the four epidemiological studies investigate the correlation between biomass process exposures and health conditions that are not strictly connected to the phases of a biogas plant. 

#### 3.3.1. Residential Setting Studies

Of the two studies carried out in residential settings, one is a cross-sectional study from Thailand [17], which aimed at exploring the impact of air pollution from the activity of two biomass power plants on nearby residents’ health. Both plants employ the same biomass type, i.e., rice husks. This study was performed on a population of 392 residents (households aged ≥ 15 years). Exposure was determined by the distance between the residential communities and the biomass power plants. Accordingly, three groups were identified: Group 1, residents living at a distance of 0–0.5 km from the plants; Group 2, residents living 0.5–1.0 km away from the plants, and Group 3, residents living at >1 km distance who were regarded as the reference category. The households reported their own health symptoms during the past week and answered questions about chronic diseases for each member of the household, resulting in 1254 participants. People working in the plants were excluded from the survey.

Air quality was measured by means of air monitoring stations; the pollutants considered were dust, total suspended particulate (TSP), particulate matter with a diameter less than 10 µm (PM_10_), nitrogen dioxide (NO_2_), sulphur dioxide (SO_2_), and ozone (O_3_). The outcomes were represented as chronic diseases (allergy, asthma, heart disease, chronic obstructive pulmonary disease (COPD), tuberculosis, and cancer) and health symptoms (itching/rash, eye irritation, cough, stuffy nose, allergic symptoms, sore throat, and difficulty breathing). The results showed (Table S1) that living near the biomass power plants was significantly associated with all health symptoms for residents living at a distance of 0–0.5 km. For the second level of exposure (i.e., residents living at 0.5–1.0 km from the plants) the relationship was statistically significant only for difficulty breathing (detailed results are shown in Table S1). With regard to the relationship between “living near biomass power plants and chronic diseases”, the only significant association was related to allergy for the first exposure group when compared to the reference group. This study has some limitations: First, it is unclear if a standardized questionnaire was used to collect data about health symptoms, which presents a risk of information bias. Secondly, the study relied on self-reported information about chronic diseases and health symptoms, which were not confirmed by a clinician and might have been subsequently overestimated. Thirdly, in reporting their symptoms, the subjects might have demonstrated a recall bias. Finally, the analyses were conducted without taking into account possible confounders.

The other study is an up-to-date cross-sectional study conducted in New York State, USA [18], which aimed at assessing the potential connection between residential proximity to biorefineries or biorefinery-related emissions and respiratory morbidity. The study population was represented by New York State (NYS) residents aged 1–85 living within 20 km from biorefineries (regarded as “biorefinery sites”) and from the reference areas who received Emergency Department (ED) visits (n = 547,437) for lower respiratory diseases between January 2011 and December 2015. The study areas included 15 biorefineries located in NYS and using different biomass types: corn (n = 2), soybean (n = 2), and wood (n = 11). Fifteen reference areas with no biorefineries were also identified. These areas shared similar socio-demographic characteristics with the biorefinery sites (median income, age distribution, and percentage of African Americans). The outcomes were lower airway diseases, including asthma, chronic bronchitis, emphysema, and chronic airway obstruction. Exposure was represented by both residential proximity to biorefineries and air dispersion-modelled concentrations of multiple pollutants. Regarding proximity, distances from refineries were expressed as 0–5, >5–10, >10–15, and >15–20 km. In a second phase, analyses were carried out within 10 km, with this distance being identified as a threshold of health risk. Among the air pollutants, particulate matter with a diameter less than 2.5 µm (PM_2.5_), SO_2_, and NO_2_ were considered. The daily concentrations of air pollutant were estimated by using the American Meteorological Society and the U.S. EPA (AMS/EPA) Regulatory Model (AERMOD) over the period between 2011 and 2015. Possible confounders (i.e., age, race, sex, county-level smoking rate, meteorological variables, and annual mean concentrations of air pollutants) were taken into account in the analyses. The results show the importance of taking SO_2_ and NO_2_ into consideration for future studies, given their contribution to respiratory ED visits. Additionally, residential proximity (within 5 km) to plants emitting PM_2.5_, SO_2_, and NO_2_, such as biorefineries, was associated with an increased risk of emphysema. The health risk seemed to be greater for residents living near corn and soybean biorefineries than for those living close to wood biorefineries; the average PM_2.5_ and NO_2_ emission rates (g/s) from corn and soybean biorefineries were 1.5−3 times higher than the average PM_2.5_ and NO_2_ emission rates from wood biorefineries. Associations between biorefinery activity and respiratory health were higher during spring and winter compared to those during fall and summer (with the lowest risk). The detailed results are shown in Tables S2 and S3. This study presents some limitations: only severe cases were considered, leaving aside mild cases; the study population included only health insurance owners; a selection bias might exist due to the geographical locations of the study population (residents of lower socio-economic status); unmeasured confounders, such as indoor exposure and pattern activity, might have introduced confounding bias; and the results might show ecological fallacy because of the aggregated nature of the data.

Table 2 shows the main features and the results of the two included studies in residential settings.

#### 3.3.2. Occupational Setting Studies

Both studies related to occupational settings were carried out in Denmark [19,20].

The study by Schlünssen et al. (2011) [19] focused on a possible association between working in a biomass-fuelled plant and an increased risk of respiratory diseases compared to working in a conventional power plant. This cross-sectional study was conducted on 85 Danish heating and power plants using woodchip or straw and 11 heating and power plants using conventional fuel. In both types of facilities, the workers had similar tasks. The study investigated the exposure to dust, endotoxins, cultivable fungi, and *Aspergillus fumigatus* (Fresenius 1863). The workers were divided into three exposure groups (most, moderately, and least exposed) based on personal mean exposure to bioaerosols, as measured through job-exposure matrices. The data on respiratory diseases were collected through the European Community Respiratory Health Survey (ECRHS) short questionnaire, in which further questions about allergy, cough, asthma, rhinitis, smoking, toxic pneumonitis, and occupational history were added. The questionnaire was self-administered to the workers. Smoking and atopy were regarded as confounders. The results showed that working either in a heating or in a power plant using biofuel did not expose the workers to any considerable additional risk for respiratory diseases; however, the level of micro-organisms (*Aspergillus fumigatus*) had an impact on the occurrence of respiratory symptoms among the biofuel workers, despite a low exposure level. It follows that preventive precautions should be taken in power plants using biofuel to keep bioaerosol exposure as low as possible (detailed results are shown in Table S4). This study presents the following limitations: the low response rate (59%) among the reference workers, especially within the least exposed reference group, might be a source of differential misclassification; respiratory diseases were self-assessed with subjective single questions; and the self-reporting of data on respiratory diseases might produce information bias.

The study by Basinas et al. (2012) [20] addressed endotoxin exposure in diverse occupational settings, combining the results of four studies carried out in Denmark and the Netherlands. For the scope of our research, we deal with the first of the four studies: a cross-sectional study performed on Danish biofuel plant workers (n = 176) aimed at exploring the relationship between bioaerosol exposure and allergy and respiratory symptoms in plants using either woodchip or straw. Four exposure groups were identified: low, low mediate, high mediate, and highly exposed (Table 3). The data about health outcomes were collected by means of a self-reported questionnaire. The confounders were gender, age, farm childhood, atopic predisposition, and smoking habits. The results showed that endotoxin exposure was statistically significantly associated with chronic bronchitis for the moderately exposed group and with wheezing for the most exposed group (detailed results are shown in Table S5). This study has some limitations: respiratory diseases were self-assessed with subjective single questions, and the self-reporting of data on respiratory diseases might produce information bias.

### 3.4. Environmental Monitoring Studies

The studies included in the environmental monitoring section reported the results of on-field measurements with quantitative assessment of emissions associated with biogas production, or with technologies with processes that are partially overlapped with biogas production ones. As shown in Table 4, it is possible to identify three main levels of monitoring:

1. Occupational level (n = 10) reporting analyses dealing with the potential exposure and risk assessment for workers;
2. Community level (n = 1) aiming to analyse air quality around biogas plants and to identify potential atmospheric pollution that can be released by biogas plants according to the context;
3. Other emissions (n = 4), including two Life Cycle Assessment evaluations that identify green gas house emission and acidification potentially associated with biogas plants, and two studies about the features of digestate.

Most of the analysed plants produce biogas through the anaerobic digestion process that, in some cases, is coupled with energy production using biogas in a co-generator. Moreover, these plants adopt different feedstocks that can be classified into four categories, as reported in Table 5: crop residues (CR), organic fraction of municipal solid waste (MSW), animal manure (AM), and wastewater sludge (WWS).

Considering the process of biogas production shown in Figure 1, Table 6 summarises the monitoring approach of the included works, including identifying the monitored phases, the approach for the sampling measurements, and the feedstocks employed in the plants, where applicable.

Regarding the contents of the analysed papers, the authors decided to organize the results according to the type of monitored pollutants:

4. Identification of biological pollutants in biomass and/or in aerosol;
5. VOCs and emitted gases;
6. Particulate matter and nanoparticles.

In addition, a fourth section reports the results of the LCA and digestate analysis.

#### 3.4.1. Biological Pollutants in Biomass and/or in Aerosol

Despite numerous benefits related to the use of anaerobic digestion as an alternative source of energy, the handling of biomass could potentially create a biological risk due to exposure to harmful microbiological agents, which could cause respiratory problems.

Selected studies found several groups of bacteria and fungi in the air near different areas of biogas plants. These organisms came from different types of biomasses (e.g., forest and agricultural residues, sludge from wastewater treatment plants, and food waste). Any differences found in the exposure levels among the plants might partly be due to differences in the process equipment, tasks, and biofuel handled.

Since the same type of biomasses may be used in both biogas- and biomass-powered plants, studies conducted in both kinds of plants were included (Table 6).

Madsen (2006) conducted a monitoring on workers’ exposure to organic dust in different working areas (e.g., storage areas and offices) of five biofuel plants in Denmark, which employed bark chips with salt water, straw, forest chips, and industry chips (Table 6) [21]. GSP inhalable samplers were mounted with Teflon filters (pore size 1 mm) for endotoxin and NAGase (N-acetyl-beta-d-glucosaminidase, an enzyme mainly produced by fungi) analysis, and with polycarbonate filters (pore size 1 mm) for quantification of total number and cultivable units of bacteria and fungi. In addition, outdoor references were sampled upwind of the plants on each sampling day. Personal exposure to inhalable endotoxins resulted in a range between 2 and 119,000 EU/m^3^ (EU: endotoxin unit), and 34% of the measured subjects were exposed to >150 EU/m^3^. The highest exposure was found for an employee who worked with a straw shredder for 90 min out of 6 working hours, while the lowest exposure was found for a worker working partly in an office and partly as a painter. In the working areas, the median endotoxin concentration was 66 EU/m^3^ (average = 429; max = 21,000; and n = 88). Personal exposures to concentrations > 104 cfu/m^3^ of mesophilic fungi were found for 81% of the workers, and 68% of them were exposed to concentrations of fungal spores > 105 m^−3^. *Aspergillus fumigatus*, a potential pathogenic fungus species, was found in most areas, and the highest exposure was found for a person who cleaned a chip pit for 1 out of 7 working hours and did office work for the remaining 6 h.

In a Finnish plant using kitchen waste for digestion (Table 6), Tolvanen and Hanninen (2006) determined the concentrations of mesophilic and thermophilic bacteria, fungi, and actinomycetes, including both viable and non-viable micro-organisms, by using a six-stage impactor and by collecting airborne micro-organisms using nucleopore filters via an estimation and analysis (CAMNEA) filter collection method [23]. They sampled five locations and activities: (I) pre-treatment and crushing of waste; (II) maintenance work in the pre-treatment hall; (III) maintenance work in the bioreactor hall; (IV) bioreactor hall during normal operation; and (V) drying hall. The level of microbes and endotoxins was a problem in the plant, especially during waste crushing, and the authors suggested the use of a respirator mask for the workers during dusty working phases. Nevertheless, the occupational hygiene of the plant was as good as that of plants treating biowaste aerobically.

Madsen et al. (2009) carried out a monitoring of organic dust in 14 Danish biofuel plants, with one plant mainly using straw and wood chips (Table 6) [25]. Twenty-nine samples of airborne particles were taken using a Triplex cyclone. Moreover, they sampled total dust using 25 mm closed-face cassettes. They assessed the concentration of microorganisms in PM_1_ dust and fungi in total dust using a modified CAMNEA method. This sampling was performed in the straw storage room at 1.5 m above floor level (i.e., the workers’ breathing zone). Cultivable mesophilic fungi and “total fungal spores” were found in 6 and 22 of the 29 samples, respectively. However, the median concentration of cultivable fungi was below the detection level, and no thermotolerant fungi were found. Moreover, for the first time, the authors found high concentrations of β-d-glucan (a polymer produced by most fungi and with a possible negative impact on health) in PM_1_ dust sampled from the straw storage room where the workers handled straw.

Ławniczek-Wałczyk et al. (2012) investigated exposure to harmful microbiological agents during the handling of biomass in a Polish power plant in which forest and agricultural biomass is co-combusted with pulverised coal (Table 6) [31]. Ten sampling stations were designated, including workplaces in the technological line (seven stations), a control laboratory where biomass quality was tested for its physical parameters (two stations), and one “background” station, to assess the reference baseline. Moreover, the measuring instruments were placed at the workers’ breathing zone height, and the microorganisms isolated from the air samples were identified down to the genus and/or species level. The concentration of bacterial aerosol ranged from 5.1 × 10^2^–2.3 × 10^4^ cfu/m^3^ in the workplaces in the technological line to 5.6 × 10^2^–3.3 × 10^3^ cfu/m^3^ in the laboratory, while the fungal aerosol concentration varied from 2.2 × 10^2^–2.0 × 10^4^ cfu/m^3^ in the workplaces to 1.1 × 10^3^–8.0 × 10^3^ cfu/m^3^ in the lab. A significantly higher concentration of bacteria and fungi in the air in the workplaces in the technological line, compared to those in the laboratory, was found. In general, the results of this study indicate that both workers employed in the technological line for biomass combustion and laboratory workers were exposed to bioaerosols containing potentially pathogenic bacteria and fungi. The qualitative analysis of the air microorganisms in the designated workplaces indicated the presence of bacterial and fungal specimens that, according to the Ordinance of the Polish Minister of Health, are classified into risk group 2, which means a possible adverse health effect (e.g., allergic reaction) for people with an impaired immune system.

Traversi et al. (2015) evaluated airborne exposure among anaerobic digestion workers at two Italian plants (S–plant and M–Plant) [27]. These plants used both agricultural and livestock biomasses (Table 6). Microbiological sampling was performed during the input and output operations of both plants, and eight variables were measured: total bacteria as an environmental contamination indicator; total bacteria as an animal/human contamination indicator; total thermophilic bacteria; fungi and yeasts; Pseudomonadaceae as a biofilm formation indicator; Clostridia spp. as an indicator to evaluate possible anaerobic digestion selection; Enterobacteriaceae as a gut contamination indicator; and Actinomycetes as another environmental microbiological component most likely linked to the investigated biomasses. Moreover, the authors sampled endotoxins to evaluate the concentration level of this component of breathable particulate. The authors found that environmental total bacteria were in the range of those for mesophilic bacteria observed in composting facilities and were equal to 10^2^–10^8^ cfu/m^3^, and the same evidence was observable for thermophilic Actinomycetes and moulds. Endotoxin concentration ranged from 5 to 3220 EU/mg, with a mean value of 428 EU/mg, which was a low range compared to those observed in other waste collection and treatment plants. In conclusion, the authors asserted that biological risk has to be carefully quantified and managed, particularly in indoor environments, and additional specific assessments may be necessary for emerging pathogens, such as viruses.

Laitinen et al. (2016) carried out occupational hygiene measurements at three biofuel-powered plants (Table 6) in Finland in order to measure the exposure of employees to biological agents [30]. They collected air samples during the unloading and processing of solid biofuels, and in the control room and outdoor (for a reference baseline). They collected both viable bacteria and fungi and biologically active endotoxins in the workers’ breathing zone. The authors found that, while unloading biomasses, the workers were highly exposed to organic dust, which was mainly composed of bioaerosols, and a high concentration of endotoxins was found. The concentrations of airborne endotoxins often exceeded the limit value of 90 EU/m^3^ (suggested by the Nordic and Dutch Expert Group), which might cause adverse health effects for workers over short- or long-term occupational exposure. Moreover, microbial concentrations during unloading operations were higher than 10^4^ cfu/m^3^, which might be a threat to the workers’ health. The authors concluded that more attention should be paid to health and safety issues when developing a bioenergy supply chain, and employee exposure should be diminished by means of technical solutions and use of appropriate protective clothing and respirators.

Ławniczek-Wałczyk et al. (2017) monitored bacterial pathogens in workplaces in a Polish biomass-powered plant fed by wood chips and agricultural waste (Table 6) [32]. The sampling points were in the workplaces associated with reloading, separation, sizing, storage, and conveyor’s transport of biomass. In each of the workplaces, bioaerosol, used respiratory masks, and swab samples from the workers’ hands were collected. The bacterial aerosol concentrations ranged from 5.6 × 10^4^ to 4.1 × 10^6^ cfu/m^3^. The highest concentrations were recorded in the workplaces involved in the conveyor’s transport of biomass, while the lowest concentrations were noted at the background sampling points. The number of bacteria in the swabs and respiratory masks reached a high value of 1.5 × 10^4^ cfu/mL and 1.9 × 10^3^ cfu/cm^2^, respectively. Moreover, the authors suggested the use of polymerase chain reaction (PCR)-based methods as a tool for a fast and precise typing of bacterial strains isolated from different sources in an occupational environment in order to implement appropriate prophylactic procedures and minimize the transmission of infectious agents at work sites

Mbareche et al. (2018) assessed fungal bioaerosols in two biomethanisation facilities (BF) in Quebec, Canada, using a next-generation sequencing approach combined with real-time PCR [33]. These plants employed both water sludge and organic industrial food waste as feedstocks for anaerobic digestion (Table 6). Air samples were collected during work activities potentially associated with aerosol exposure at several plant sites (e.g., reception, biomass treatment area, and storage hall). An outdoor control air sample was also collected outside each plant as the reference baseline. The authors focused their study on *Penicillium*/*Aspergillus* spp. and *Aspergillus fumigatus* because of their potentially hazardous effects. The results are reported as number of ITS genes/m^3^, i.e., genic sequences belonging to the internal transcribed spacer of the selected fungus specimens. The concentrations of *Penicillium*/*Aspergillus* spp. ranged from 6.4 × 10^2^ to 1.2 × 10^4^ ITS genes/m^3^. All measured concentrations were higher in the summer than in the winter for both plants. For BF1, the reception, storage, and output sites had the highest concentrations of *Penicillium*/*Aspergillus* spp. (10^4^ ITS genes/m^3^). For BF2, similar concentrations were noted at the reception/shredding site and were higher than the concentrations identified at the mixing site. *Aspergillus fumigatus* was detected in the samples from all sampling sites in both facilities. The concentrations ranged from 9.6 × 10^1^ to 1.2 × 10^4^ ITS genes/m^3^. Comparisons between the facilities during the summer and winter showed trends that were similar to the *Penicillium*/*Aspergillus* spp. results. The highest concentrations (10^3^ ITS genes/m^3^) of *Aspergillus fumigatus* were found in the storage and output sites in BF1 during summer. For BF2, the reception/shredding site had the highest concentration (1.2 × 10^4^ ITS genes/m^3^) of *Aspergillus fumigatus* during summer, but a lower concentration (2.7 × 10^2^ ITS genes/m^3^) during winter. The differences between the concentrations of *Penicillium/Aspergillus* spp. and *Aspergillus fumigatus* from each sampling site were approximately one log, which indicates a dominance of *Aspergillus fumigatus* in the bioaerosol samples. The outdoor control samples collected during each visit were below the detection limit, indicating a very low presence of fungal bioaerosols in the air. The broad spectrum of fungi detected in this study included many known pathogenic agents, and the authors found fungal signatures associated with the type of waste treated. This study highlighted the importance of using a high-throughput sequencing method combined with a real-time qPCR assay for quantification and an in-depth characterization of fungal diversity in bioaerosols to assess occupational exposure. Moreover, the authors strongly recommended taking action to monitor workers’ exposure to these aerosols. Better air exchange rates, better confinement, and source ventilation may be suitable organizational measures to limit workers’ exposure. Otherwise, skin and respiratory protection for workers may be useful to reduce continuous exposure to harmful fungi present in bioaerosols.

Traversi et al. (2018) analysed workers’ bioaerosol exposure in several Italian plants that used agricultural and livestock biomasses (n = 3); food and feed producing by-products and food waste (n = 1); and wastewater sludge (n = 1; Table 6) [29]. Bioaerosol sampling was performed in these plants, and twelve microbiological variables were measured (bacterial environmental total count at 22° C; bacterial total count at 37 °C; thermophilic total count at 55 °C; yeasts/fungi; Pseudomonadaceae; *Bacillus* spp.; Clostridia; Gram-negative bacteria; *Salmonella* spp. and *Shigella* spp.; Actinomycetes; Enterobacteriaceae; and *Staphylococcus* spp.). In decreasing order, the authors found staphylococci, bacilli, enterococci, and Clostridia. Moreover, the levels of Gram-negative Pseudomonadaceae, *Salmonella* spp., and *Shigella* spp. were very limited.

The authors asserted that the management of biological risk deserves specific attention, especially in indoor areas, where organic wastes are treated. In these settings, bacterial and mould contamination is quite remarkable, and the presence of various pathogens is also shown to be dispersed into the air as part of the bioaerosol. Moreover, the maintenance technicians and workmen involved in the cleaning procedure near the biomasses showed bioaerosol exposure. The estimated occupational risk is considerable not only for bioaerosol, but also for endotoxin and particulate exposure. Nevertheless, the authors claimed that such problems cannot be an obstacle to the diffusion of anaerobic digestion treatment of organic waste and biomasses.

#### 3.4.2. Volatile Organic Compounds and Gases Emitted by the Processes

Besides the assessment of biological risk, chemical characterization of pollutant emissions during the production and use of biogas is essential for a comprehensive evaluation of potential exposures. The results identified in the literature are organized according to the scope of the measurement and the monitored phases: occupational risk analysis of VOCs emitted during transport and handling raw materials, i.e., by-products of wood manufacturing and wastewater treatment [30]; occupational risk analysis of VOCs emitted by the anaerobic digestion process, i.e., food waste processing [34]; and environmental analysis of gas and particulate concentration by agricultural waste and biomasses [22].

Laitinen et al. (2016) reported a detailed personal monitoring of VOCs, dusts, and biological pollutants, focusing on workers responsible for transporting and unloading raw materials from trucks [30]. Although in a facility implementing a different technology, the results of the analysis are also relevant for biogas plants processing the same biomasses (i.e., forest chips, whole-tree chips, stem wood chips, wood processing industry residues, stumps, bask, wastewater sludge, and milled peat) since transport and handling of raw feedstock are included in the process. They demonstrated that employees were exposed to a huge amount of organic dusts and VOCs during the unloading of indigenous fuels (i.e., biomasses processed by the plant). The highest measured concentration of total VOCs during the unloading of fuels was six times the reference value recommended by the Finnish Institute of Occupational Health (18,000 mg/m^3^ vs. 3000 mg/m^3^). Table 7 shows the breakdown of the main detected VOCs, which were identified as being originated from pine and spruce softwood: α-pinene, Δ3-carene, β-pinene, limonene, and other monoterpenes (C_10_H_16_). Moreover, an analysis of the reception hall highlighted a significant concentration of acetone and sulphur dioxide, which, according to the authors, was due to the emissions from trucks powered by diesel gasoline. As a conclusion, the authors highlighted the main work tasks that possibly caused exposure, namely unloading, screening, crushing, conveying of fuels, and handling of biomass in silos.

Zheng et al. (2020) analysed VOCs emitted by a plant with a treatment capacity of 300 tons/day of food waste [34]. The sampling points corresponded to four main working units: the sorting/crushing room (SR), the hydrothermal hydrolysis unit (HH), the anaerobic digestion unit (AD), and the biogas production unit (BP); different seasons were analysed along the one-year monitoring period. Table 7 summarises the main results of the monitoring. In particular, the analysis showed the highest cumulative emissions in spring (9.54 × 10^4^ μg/m^3^), while the lowest was in winter (1.39 × 10^4^ μg/m^3^), depending on the outdoor conditions and the composition of the feedstock. The working units showed different VOC concentration, and in particular, the HH unit presented a concentration significantly higher than other sampling points (3.49 × 10^4^ μg/m^3^), followed by the SR unit (8.97 × 10^3^ μg/m^3^), the anaerobic digestion unit (6.21 × 10^3^ μg/m^3^), and the biogas production unit (2.01 × 10^3^ μg/m^3^).

With regard to environmental analysis, the study by Merico et al. (2020) aimed to assess the potential influence of biogas plant emissions on local air quality, focusing on a plant for the production and combustion of biogas from agricultural wastes and biomasses in Italy [22]. The monitoring took place at 100 m from the boundaries of the biogas plant, which annual production accounts for 4,106,250 Nm^3^ of biogas that is converted into energy through a co-generator working 24 h/day. Table 8 shows the overview of the main results in terms of emitted gases and particulate of the monitored sites (values in grey lines) in comparison with the limits set by the European Directive 2008/50/CE [35]. Although some significant peaks that can be attributed to the biogas plant were detected, the general results show average gas concentrations within the thresholds identified by the European Directive [35].

The authors found that vehicular traffic and biomass combustion (from agricultural activities and domestic heating) significantly influenced CO and nitrogen oxides [36], while NO emissions could be ascribed to the biogas production and combustion plant. SO_2_ concentration was predominantly due to transport from a nearby industrial zone. However, a second local contribution compatible with the emissions of the biogas production and combustion plant was found.

#### 3.4.3. Digestate Analysis

Digestate is one by-product of the anaerobic digestion process, and it is often employed as a fertilizer in agriculture due to its high nutritional value in terms of nitrogen and phosphorous concentrations. The application of this product in agriculture should always be preceded by an analysis of its safeness from a health point of view. The two studies investigating this topic found through the database search were conducted in California in 2017 and in China in 2022.

In a Californian plant, Kuo and Dow (2017) analysed some variables of the digestate obtained from the anaerobic digestion of fats, oils, grease, food waste, and wastewater sludge [24]. They found that all these variables were in the narrow ranges; thus, the anaerobic digesters of this plant could be considered as operating under stable conditions. Moreover, the ammonium concentration (1137 ± 83 mg/L) did not seem to inhibit biological activities.

In 2022, Ke and colleagues investigated the composition of digestate produced from 5 different feedstocks collected from 31 biogas plants in China [28]. More specifically, thirteen plants used swine manure as digester feedstock, seven used cattle manure, four used straw–manure mixture, four used chicken manure, and three used kitchen waste. Digestate samples were obtained from the drainage pipeline of the digesters and analysed using ion chromatography, liquid chromatography–tandem mass spectrometry, and inductively coupled plasma–tandem mass spectrometry. These analyses assessed chemical oxygen demand (COD), nitrogen and phosphorous concentrations, concentration of antibiotics and hormones, and concentration of heavy metals. Furthermore, the authors employed these analyses to calculate the eutrophication potential of digestate—combining the values of COD, nitrogen, and phosphorous—and the potential ecological risk index, using the concentration of heavy metals. The eutrophication potential of digestate was calculated for each feedstock, considering the potential of COD, phosphorous, and nitrogen. The eutrophication potential of digestate from chicken manure proved to be the most concerning for eutrophication threat, although this digestate contained the highest nutrient concentration for agricultural purposes. On the other hand, the digestate from cattle manure showed the lowest eutrophication potential. The antibiotic concentration was the highest in swine manure digestate. The risk indices calculated for heavy metal concentration showed that most digestates had a moderate potential ecological risk, except for the cattle manure digestate, which values were lower. These results indicated that the potential threat from digestate might be independent of the feedstock. The potential ecological risks of As and Hg were particularly high.

## 4. Life Cycle Assessment (LCA)

LCA deals with the estimation of potential environmental impact in terms of carbon footprint, CO_2_ emissions, and global warming potential, representing indicators that are relevant for the potential health impact of biogas generation.

The two identified studies aimed to analyse the life cycle assessment of anaerobic treatment of human waste in the European and Chinese contexts. In particular, Iordan (2016) evaluated the impact of producing 1 MJ of energy through biogas derived from the digestion of urban waste at a Norwegian plant [10], while Duan et al. (2020) focused on the impact of an optimised anaerobic digestion process in terms of LCA and identified scenarios for energy system development in China [26].

Iordan (2016) assessed the LCA indexes of a process for treating the quantity of human waste required to generate 1 MJ of electricity through a CHP [10]. The main calculated indices were Global Warming Potential (GWP) with a time horizon of 20 and 100 years, and Global Temperature Change Potential with a time horizon of 20 and 100 years. The indices were reported considering the breakdown of N_2_O, CH_4_, and CO_2_ emissions and including Near-Term Climate Forcers (NTCFs).

These species include the following pollutants: nitrogen oxides (NO_x_), carbon monoxide (CO), volatile organic compounds (VOCs), black carbon (BC), organic carbon (OC), and sulphur oxides (SO_x_). NTCFs may present a negative impact on GWP and GTP, resulting in a cooling contribution (represented as a negative value of gCO_2_–eq). The LCA for producing biogas energy and for managing digestate was then compared with the LCA for the reference scenario, based on the current energy mix in Norway [37,38]. A significant reduction in terms of CO_2_ due to energy production from biogas was identified, which, on the other hand, causes an increase in CH_4_ and NO_2_ emissions when compared to the reference scenario. This increase is associated with digestate management (mainly due to the open storage) and methane (CH_4_) losses during the anaerobic co-digestion. Moreover, terrestrial acidification potential (TAP), photochemical oxidant formation potential (POFP), and particulate matter formation potential (PMFP) were evaluated. The POFP of the biogas system is 10% lower than the reference system. On the other hand, the TAP and PMFP performance of the reference system is better when compared to the biogas system. This is mainly due to the large influence of digestate management.

This work also assessed the contribution of the different phases and the factors mainly affecting the environmental impact of a biogas plant, namely N_2_O emissions from storage, CH_4_ leakages from the anaerobic tanks, and transport distances for the four feedstock types. In particular, closed storage was the scenario with the best GWP100 result, with 43 g CO_2_–eq/MJ, which represented a 41% improvement from the base scenario. The largest contributor was the anaerobic digestion process due to CH_4_ losses, with a 33% share, followed by feedstock transport, with a 14% share, and digestate transport and spreading, with a 12% share. In this case, CO_2_ contributed to 52% of the total GWP100, followed by CH_4_ emissions, at 46%, and N_2_O, at 2%. When the transport distances for all four feedstock types were doubled, the GWP100 increased by 9%, at 79 g CO_2_–eq./MJ, with CO_2_ and N_2_O contributing at 35% and CH_4_ at 30%. This parameter was not as sensitive as CH_4_ losses or digestate storage. The analysis showed that the most sensitive parameter was digestate storage, which had the highest impact on the LCA; specifically, closed storage might be regarded as the preferable digestate management option [3].

Duan et al. (2020) analysed a process based on anaerobic digestion of human waste, which aimed at producing biogas either for electricity generation through a combined heat and power production (CHP) plant or for conversion in biomethane [26]. The initial inputs of the LCA were the evaluation of the amount of human waste available for anaerobic digestion treatment and the potential of daily electricity production that could be generated through biogas combustion in the CHP plant at the national level in China. The authors estimated that the potential energy generated by biogas from available human waste has the potential to reduce the annual emissions of around 142 kt/CO_2_–eq at the energy system level.

## 5. Discussion

This work provides a comprehensive scientific overview that outlines the topic of electricity, biogas, and heat and biofuel generation through biomass use and the resulting health impacts on humans, both in occupational and residential settings. The aim was to answer the following research question: “What are the risk factors for the health of the people working in biogas plants and of the surrounding communities?” As a matter of fact, in the diverse research fields taken into consideration, i.e., environmental monitoring, epidemiology, toxicology, and human biomonitoring, an extremely heterogeneous terminology is employed. It follows that the keywords selected to build the search strings might prove to be adequate for certain fields, but not for others, where some relevant studies might have been missed. As a result, among the four research areas that were originally considered, only two were included due to the scarce number of results displayed in the others. Additionally, due to the small number of primary studies identified through the search strings and employed according to the inclusion criteria, twelve studies were identified through citation searching. Nineteen studies were, therefore, included in the review. Among them, 10 studies focused on biogas production through the anaerobic digestion of biomasses, and 9 studies dealt with biomass combustion. 

Most of the studies were conducted in Europe, followed by North America and Asia. In regard to the four epidemiological studies, they were cross-sectional studies from Europe (Denmark, two studies), USA (New York State, one study) and Asia (Thailand, one study). Within this group, two were carried out in residential and two in occupational settings. The outcomes of interest were mainly respiratory symptoms and diseases, allergic reactions, and skin complaints.

We remind that the epidemiological studies investigated the correlation between biomass process exposures and health conditions not strictly connected to the phases of the biomass power plant. It is worth noted that the pollution (e.g., emission of S02 and NO2) and medical conditions related to the presence of the biorefinery cannot be reconducted directly to the phases that are in common with a biogas plant as well as endotoxins might be in common with a biogas plant, the respiratory and allergy conditions might be influenced by the biomass combustion activity.

The 15 environmental studies, mainly conducted in Europe, were classified into 3 categories: most of them (10) monitored power plants from an occupational point of view; one study monitored air quality at a community level; and the last category, “other studies”, consisted of two studies focusing on LCA analyses and two studies focusing on digestate analysis.

### 5.1. Epidemiological Studies

#### 5.1.1. Residential Setting Studies

Both residential setting studies analysed the impact of living near industrial facilities using biomass on residents’ respiratory diseases and health symptoms. As mentioned before, the studies by Juntarawijit (2013) and Lee et al. (2021) appeared markedly different, especially because the former was carried out in two plants producing electricity from the same biomass type (rice husks) but by means of two distinct technologies (steam turbine and gasification with an internal combustion engine, respectively) [17,18]. The latter, instead, analysed 15 biorefineries producing ethanol from three biomass types: corn, wood and soybean. Despite these striking differences, both studies focused on the association between living in the vicinity of the facilities under study and the risk of respiratory morbidity (as well as other health symptoms) for the nearby communities. Additionally, the air pollutants taken into consideration were similar: particular matter (PM_10_ by Juntarawijit and PM_2.5_ by Lee et al.), sulphur dioxide, nitrogen dioxide, and ozone only in Juntarawijit (2013) [17]. The health effects of these pollutants are often well known [39]. PM exposure has been linked to cardiovascular and respiratory negative outcomes, as well as cancer. In particular, exposure to PM 2.5 has raised wide concern regarding mortality and premature death due to both long- [40,41,42] and short-term exposures [43,44]. Ozone is an oxidative gas with strong irritative effects on airways, with proven association to hospital admission [45] and mortality [46], although the health effects of long-term exposures still remain a matter of debate [47]. Exposure to nitrone dioxide has also been observed to be linked to respiratory, cardiovascular, and metabolism-related diseases [48,49,50], resulting in an increased mortality among those exposed [46]. Moreover, exposure to sulphur dioxide seems to exert its health effects on respiratory diseases and related mortality [51].

Consistent with these observations, the results of the study from Thailand revealed that living in proximity of the two biomass power plants was associated with an increased risk of suffering from respiratory diseases and other health symptoms.

Similar results are shown in the study by Lee et al. (2021), which dealt with the health impact of living in the vicinity of biorefineries using different types of biomasses [18]. We decided to include this study in our review not only because one of the stages involved in the biofuel production is represented by biomass fermentation, but also because of the high methodological quality of the study itself. In fact, in this study, information about respiratory diseases was retrieved from objective hospital data. Additionally, the large sample size analysed increased the statistical validity of the analysis.

Secondly, the association between biorefinery exposures and respiratory diseases was investigated according to two exposure indicators, that is, residential proximity and AERMOD-modelled air pollutant concentrations, to validate the findings.

Thirdly, seasonal difference was taken into consideration in the association between residential exposure and lower airway diseases: as a matter of fact, respiratory ED visit rates among residents living within 10 km of the biorefineries were significantly higher than those living in the reference areas in spring and winter. These data contribute to the high methodological quality of the study, even though some parameters must be taken into account: (I) increasing air pollution levels during spring and winter can be due to photochemical decomposition rates of air pollutants at cold temperatures; (II) during cold seasons, there are higher air pollution concentrations due to the stagnation of air pollutants; and (III) tree and grass pollens cause respiratory symptoms, such as asthma, in spring.

In conclusion, the high-level methodology adopted by Lee et al. (2021) should be considered as a reference model for other residential setting studies: the same methodological approach may also be used for the assessment of health risk among workers in industrial facilities, with the administration of an ad hoc, standardised, and validated questionnaire to investigate participants’ lifestyle [18]. If this study has an occupational setting counterpart, a complete model about human health risk will be available.

#### 5.1.2. Occupational Setting Studies

Both studies exploring the health risks connected to biomass-based power generation in an occupational setting were carried out in Danish plants fuelled by straw and woodchips. While the study by Schlünssen et al. (2011) dealt with a generally formulated exposure to dust, fungi, airborne endotoxins, and *Aspergillus fumigatus* [19], the study by Basinas et al. (2012) only focused on endotoxin exposure [20]. 

The results of both studies confirm that exposure to endotoxins and fungi is significantly associated with respiratory symptoms. As mentioned before, due to the inherent characteristics of the biomasses in question (straw and woodchips), workers may be exposed during the pre-combustion phase to bioaerosols, including dust, microorganisms, and endotoxins, released during biomass handling, transport, storage, and agitation. Thus, the risk of bioaerosol formation should be limited, e.g., minimising storage times and avoiding conditions that favour mould development (for instance, accumulation of biomass in warm conditions).

Additionally, the association between exposure to certain fungal types, namely *Aspergillus fumigatus*, and respiratory symptoms, such as work-related asthma/wheeze and work-related rhinitis, implies the need for adequate control measures in order (I) to reduce workers exposure to fungal spores, and (II) to implement health surveillance to identify workers who may have a predisposition to health effects caused by the exposure.

Unlike traditional fuels, biomass tends to decompose, thus creating diverse exposure conditions and requiring different handling, transport, and storage methods in order to limit microbial growth (e.g., spore formation and endotoxin release), as well as emissions of VOCs or other gases. Furthermore, proactive training on biomass handling practices and health surveillance that focuses on workers’ respiratory health may represent a good starting point in that they would provide data on monitoring, occupational exposure, and risk assessment.

In conclusion, even though the environmental monitoring studies proved the harmfulness of particular matter (e.g., dust), microbial agents (e.g., fungi, bacteria), endotoxins, and VOCs in occupational settings, there is a need for epidemiological studies that focus on the effects that these substances have on workers’ health. An integrated approach combining environmental data with health data to investigate if they are significantly associated should be adopted.

### 5.2. Environmental Monitoring Studies

#### 5.2.1. Biological Pollutants in Biomass and/or in Aerosol

Biological pollutants (e.g., bacteria, fungi, and endotoxins) present in industrial biomasses can be harmful when inhaled by humans. In particular, exposure to endotoxins may represent an important threat to human health. In fact, endotoxins are high-molecular-weight lipopolysaccharides present in the outer layer of the cell wall of Gram-negative bacteria [52], with a high biological activity and an important resistance to heat. Thus, endotoxins cannot be removed by thermic treatments or disinfection procedures, resulting in respiratory diseases as well as immunotoxic effects [53] in workers exposed to them.

Thus, assessing the concentration of these pollutants in plants where biomasses are stocked and handled is useful for the protection of workers’ health. Each of the included studies monitored different biological pollutants in different sampling zones of the plant. Thus, a general summary of the results cannot be outlined. On the other hand, a few considerations can be drawn.

First of all, the CAMNEA (collection of airborne micro-organisms on nucleopore filters, estimation, and analysis) method [54] was employed in several studies.

Two studies [21,33] assessed the concentration of *Aspergillus fumigatus*, a potentially toxic fungus species. Thus, monitoring the concentration of this species and other congeneric species may lead to an improvement in the health of workers at biomass power plants.

Traversi et al. (2015) conducted a study in an attempt to provide reference guidelines [27]. In their study, they provided a list of safety limits for microbiological risk and for endotoxin inhalation (Table 9). For microbiological risk, the Global Index of Microbial Contamination (GIMC) and the index of Mesophilic Bacterial Contamination (MBC) proposed by Dacarro et al. (2000) were considered as the reference values [55]. The GIMC is calculated as the sum of the values of the total microbial count determined for mesophilic bacteria, psychrophilic bacteria, and fungi in all sampled areas. The MBC is obtained by calculating the ratio between the cfu/m^3^ value measured for mesophilic and psychrophilic bacteria in the same sampling point [55]. These indexes may be adopted in the monitoring of biological pollutants at biogas production plants. For endotoxin inhalation, two limit values were proposed, namely a punctual value by Feron et al. (1998) and a range value proposed by Duquenne et al. (2014) [56,57].

#### 5.2.2. Volatile Organic Compounds and Gases Emitted by the Processes

The literature analysis shows a limited number of studies, including an on-field monitoring of biogas plants, that are not homogeneous in terms of adopted feedstock, boundary conditions, technical process, and operation. Therefore, the results do not lend themselves to generalisation and may not be adopted as a direct reference for perfecting the processes and working procedures of each type of biogas plants. Moreover, the results cannot be adopted to exclude occupational and community potential risks that can be attributed to biogas plants. Nevertheless, the reported indications can be used as a preliminary reference for identifying key aspects to be controlled, parameters to be monitored, and conservative safety measures to be introduced for workers of a biogas plant. Long-term exposure to high concentrations of VOCs may entail a chronic or carcinogenic human health threat. Therefore, knowing the typology of VOCs and quantifying their emission are essential for adopting the needed control measures to reduce the potential impact on workers and communities.

Although the number of analysed works is limited, some interesting considerations can be derived as preliminary inputs of the potential risks to be evaluated in a biogas plant and promising fields for further investigations.

In fact, there are some common VOCs identified by both studies dealing with VOCs in occupational settings [30,34] despite the adoption of different feedstocks, which were wooden by-products and food waste, respectively. One of the common VOCs is limonene, which is classified by the European Chemical Agency (ECHA) as a skin sensitizer, causing skin irritation and possible allergic skin reaction. Although it is not classified as a cancerogenic substance for humans, the ECHA highlights that limonene presents a property of concern for chronic exposures. This information is important, especially for controlling the potential exposure of workers, whose skin may be in contact with limonene during the handling of both raw feedstocks and products of anaerobic digestion.

Another important result from Zheng et al. (2020) is the detection of high concentration of 1,2-dichloroethane during the process, especially during anaerobic digestion [34]. The ECHA has classified this compound as a “substance of very high concern” for both short-term and long-term exposure. In fact, on the one hand, it is harmful if swallowed and toxic in case of inhalation, and it may cause serious eye and skin irritation and respiratory inflammation. On the other hand, it has a possible carcinogenic effect, being classified in the group 2B by the International Agency for Research on Cancer. Therefore, the release of 1,2-dichloroethane during anaerobic digestion needs to be monitored and effectively controlled for the safety of the operators and in all the phases of the process. This would also be important for avoiding potential impacts on the community. In fact, the analysis of cancerogenic risk in Zheng et al. (2020) was performed by measuring the pollutant concentration at the plant boundary to identify the potential threat to the nearby community [34]. The results exceed the safety threshold of negligible cancer risk and indicate that the monitored release of VOCs from the analysed plant can threaten the health of the surrounding communities.

In regard to environmental analysis, the study by Merico et al. (2020) presented a comprehensive overview of the potential impact of a biogas plant processing agricultural waste at the community level, with a detailed analysis for apportioning the contribution of different sources (e.g., other surrounding industries, traffic, and heating systems) [22]. The monitoring shows gaseous (CO, NO, NO_2_, NO_x_, SO_2_, and O_3_) and particle emission values that are below the annual thresholds provided by the Directive 2008/50/CE (Table 8). Considering the annual average (calculated by extrapolating the results from the monitoring performed during the period 30 January–28 March 2018), the measured values do not exceed 50% of the limit values, for both the environmental limits to the more restrictive reference values for human health, namely ranging from 1% for SO_2_ to 42% for NO_x_. On the other hand, the hourly monitored values are closer to the thresholds of the Directive 2008/50/CE, reaching 64% of the environmental limit for ozone, while for the limits for human health, the values are 55% for CO and 71% for NO_2_. The results of this study cannot be adopted as a reference for all biogas plants processing agricultural waste since the environmental analysis is related to the specific features of the plant, in particular to the context and the meteorological conditions. Nevertheless, this work demonstrated that, under specific conditions, the impact of a biogas plant can contribute to a significant increase in gaseous concentration in the environment, which can reach the thresholds indicated in the Directive 2008/50/CE. Therefore, the characterisation of gaseous emissions from a biogas plant would require on-field monitoring.

#### 5.2.3. Digestate Analysis

The number of studies addressing the analysis of digestate was very low (n = 2). Nevertheless, these two studies provided useful information about the usage of digestate produced by the fermentation of different biomasses (e.g., agricultural and livestock residues, and kitchen waste).

The usage of digestate should be promoted as a positive reuse of resources, but strict health and environmental controls must be made.

The two analysed studies reported different conclusions. In the Californian plant [24], the only substance monitored in the digestate—ammonium—showed a low concentration, which could not inhibit biological activities. On the other hand, in the Chinese plants [28], the digestate was analysed for several substances, ranging from antibiotics to heavy metals, in order to assess the quality and health security of the digestate. The use of this digestate did not guarantee health nor environmental security, but the peculiarity of the study context, including the origin of the biomasses and the Chinese regulation on these topics, does not allow a comparison between this kind of digestate and the one produced in EU plants, where digestate use is regulated by the Commission Regulation (EU) No142/2011.

From an environmental point of view, the digestate produced by chicken manure should be used in agriculture, considering its very high eutrophication potential.

#### 5.2.4. LCA Analysis

The evaluation of the LCA indicators does not provide a direct measure of pollutants emitted by a biogas plant, since the assessment is not based on direct monitoring but calculated according to standard reference procedures. Nevertheless, these indicators represent an indirect quantification of the potential impact on workers and on surrounding communities, providing estimations of the global warming potential and the pollutant emissions that can be associated with biogas production. The selected works analysed the life cycle of the energy production through biogas, from the transport of raw material to the co-generator and the inlet in the electrical grid, and compared the results with the traditional energy mix of the country under study. As a general consideration, the adoption of biogas for electric power generation would allow a significant reduction in CO_2_ emissions. On the other hand, Iordan (2016) highlighted a significant increase in methane emissions derived from the anaerobic digestion, which needs to be addressed to reduce the negative impact [10]. The authors recommended reducing these emissions by means of direct interventions on the leakages of anaerobic digestion tanks. In conclusion, despite the adopted selection process identified in the two studies, the outlooks implied by the results highlight that electricity production through biogas has a positive impact in terms of LCA and reduction in CO_2_ emissions.

## 6. Conclusions

This literature review highlights that the available knowledge on the potential occupational and community hazards associated with biogas plants is fragmented. On the one hand, there is a limited number of studies including on-field monitoring due to the novelty of the technology. On the other hand, the heterogeneity of biogas plants in terms of processed feedstock, technical features of the monitored sites, specificities of the national standards, and differences from other bioenergy technologies do not enable a direct comparison among different studies. Therefore, the results cannot be used to define consistent benchmarks but provide an overview of different aspects of biogas generation, with the purpose of stimulating the discussion on this technology both at the scientific and policy levels.

Workers and populations living near biogas plants could be potentially exposed to different pollutants, namely PM_10_ and PM_2.5_, ozone, nitrogen dioxide, sulphur dioxide, some bacteria and fungi, endotoxins, and some VOCs. 

The health effects of short-term exposures to PMs, ozone, nitrogen dioxide, and sulphur dioxide are generally well known, although the effects of long-term exposures are not always completely understood. Thus, environmental monitoring should not be neglected in these populations.

Some health issues could also arise from microbiological exposure to bacteria, fungi, and endotoxins. In particular, endotoxins could expose workers to some health risks, considering their high biological activity and thermic resistance.

Even though VOCs are compounds which toxicity is generally well known, some exposures could occur in biogas plant workers, and thus, environmental monitoring should not be neglected.

Through environmental measurements, the monitored biogas plants included in the study highlighted the release of VOCs, gaseous pollutants, and particulate. This may be due to the substances used in the process. In certain cases, the control of the emissions of potential hazardous VOCs may be necessary. The following compounds have been detected in the plants analysed in the literature: 1,2-dichloroethane, benzene, ethylbenzene, 1,1,2-trichloroethane, tetrachloroethylene, styrene, tetrachloromethane, and limonene.

Monitoring requires real-time measurement and aims to identify the boundary conditions and phases, as well as the settings characterised by the highest pollutant release. This monitoring allows the creation of background knowledge that can be used to improve the management of biogas plants and control emissions. Intervention in biogas plants’ technical features is regarded as a solution to reduce pollutant emissions. Furthermore, the main outcome of the literature analysis is that methane emissions can be diminished by controlling the leakages of anaerobic digestion tanks, a simple intervention that can be easily replicated.

In regard to the epidemiological analysis, the studies highlighted the importance of setting up a health surveillance program on workers and residents regarding their lifestyle (smoking, physical activity, diet, alcohol consumption, and health status) in order to effectively monitor the potential effects of occupational exposure in biogas plants.

With regard to the biological risk, monitoring sessions of airborne micro-organisms in different work areas of biogas plants through the CAMNEA method should be programmed. Finally, the monitoring of the eutrophication potential of digestate produced by different feedstocks, starting from sample plants, and the control of heavy metal concentration in digestate should be promoted.

## Figures and Tables

**Table 1:** Key aspects taken into consideration in the review.

Focus	Technology	Topic	Exposed Subjects
Health/human	Biomass/biogas/anaerobic digestate/biofuel	Hazard/risk/exposure/impact	Worker/resident/community/population

**Table 2:** Main features of the included epidemiological studies in residential settings.

	Main Features of the Study by Juntarawijit (2013) [17]	Main Features of the Study by Lee et al. (2021) [18]
Study population	Total of 392 residents: 181 living near Plant I (steam turbine technology), and 211 living near Plant II (gasification technology and internal combustion engine) in Thailand.	NYS residents aged 1–85 living within 20 km from biorefineries and in the reference areas who had ED visits (n = 547,437) for lower respiratory diseases during the period January 2011–December 2015. Study areas: 2 corn biorefineries, 2 soybean biorefineries, and 11 wood biorefineries located in NYS. Reference areas: 15 sites with no biorefineries located in NYS.
Exposure	Living near biomass power plants. Exposure Group I: 0–0.5 km; II: 0.5–1.0 km; and reference group: >1 km.	1. Residential proximity to biorefineries [straight-line distances expressed in km (0−5; >5−10; >10−15; and >15−20 km)]; 2. air dispersion-modelled concentrations of multiple pollutants (PM_2.5_, SO_2_, and NO_2_).
Outcomes	Chronic diseases (allergy, asthma, heart disease, COPD, tuberculosis, and cancer) and health symptoms (itching/rash, eye irritation, cough, stuffy nose, allergic symptoms, sore throat, and difficulty breathing).	Lower airway diseases (asthma, chronic bronchitis, emphysema, and chronic airway obstruction).
Data sources	Data on chronic diseases and health symptoms: self-reported questionnaire; air quality measured by air monitoring stations (dust, TSP, PM_10_, NO_2_, SO_2_, and O_3_).	1. SPARCS database (respiratory hospital ED visits); aggregated number of ED visits due to the following respiratory diseases: asthma, chronic bronchitis, emphysema, and chronic airway obstruction. 2. AERMOD dispersion model (concentration of air pollutants). 3. U.S. EPA (seasonal mean temperature and relative humidity and annual mean air pollutant concentrations).
Biomass type	Rice husks	Corn, soybean, and wood
Production	Electricity generation	Biofuel
Health symptoms	Chronic diseases (allergy, asthma, and COPD), allergic symptoms, cough, difficulty breathing, eye irritation, itching/rash, sore throat, and stuffy nose	Asthma, chronic bronchitis, emphysema, and chronic airway obstruction
Exposure level measured by	Plant proximity	Plant proximity
Outcome identification	Self-reported questionnaire	Hospital data
Results	There is an association between living in the vicinity of the two biomass power plants and the aforementioned respiratory and health symptoms	Respiratory ED visit rates among residents living within 10 km of biorefineries were significantly higher than those living in the reference areas according to residential proximity and air pollutants. This relationship considered biorefinery types, seasons, air pollutant types, and respiratory subtypes (highest for emphysema)

**Table 3:** Main features of the included epidemiological studies in occupational settings.

	Schlünssen et al. (2011) [19]	Basinas et al. (2012) [20]
Study population	232 energy plant workers: 94 straw workers, 138 woodchip workers, and reference group (107 working in a conventional power plant) from Denmark.	176 biofuel workers (woodchip and straw) from Denmark.
Exposure	Working in a (straw or wood) plant. Exposure to dust, airborne endotoxins, cultivable fungi, and *Aspergillus fumigatus*. Personal mean exposure to dust, endotoxins, and cultivable fungi (3 levels: low, medium, and high).	Endotoxin exposure in a straw/wood power plant. Four exposure groups: low (<50 EU/m^3^), low mediate (50–200 EU/m^3^), high mediate (200–1000 EU/m^3^), and highly exposed (>1000 EU/m^3^); the lowest exposure group taken as the reference group. Median estimated average endotoxin exposure: 0.01–294 EU/m^3^.
Outcomes	Respiratory diseases (asthma symptoms, current asthma, rhinitis, chronic bronchitis, work-related asthma/wheeze, and work-related rhinitis symptoms).	Asthma, chronic bronchitis, hay fever, allergy, organic dust toxic syndrome, wheezing, and atopy.
Data sources	Data on respiratory diseases: ECRHS study; stationary work areas measurements (work areas surveyed: boiler room, combined reception and storage hall, repair room, office, outdoor work, and weighing room).	Data on respiratory diseases: self-reported questionnaire; stationary dust samples collected in all working areas.

**Table 4:** List of included papers identified as environmental monitoring studies. The papers are listed chronologically.

Occupational Level	Community Level	Other Emissions
Madsen, 2006 [21]	Merico et al., 2020 [22]	Iordan, 2016 [10]
Tolvanen and Hanninen, 2006 [23]		Kuo and Dow, 2017 [24]
Madsen et al., 2009 [25]		Duan et al., 2020 [26]
Traversi et al., 2015 [27]		Ke et al., 2022 [28]
Traversi et al., 2018 [29]		
Laitinen et al., 2016 [30]		
Ławniczek-Wałczyk et al., 2012 [31]		
Ławniczek-Wałczyk et al., 2017 [32]		
Mbareche et al., 2018 [33]		
Zheng et al., 2020 [34]		

**Table 5:** Overview of the plants analysed in the included studies (BG= biogas plants implementing anaerobic digestion, and BC= power generation through biogas combustion).

Reference	Type of Plant	Classification of Adopted Feedstock				Detailed Description of Feedstock as Reported in the Paper	Plant Size (Generation Capacity/Amount of Feedstock Processed)
Title		CR	MSW	AM	WWS		
Duan et al., 2020 [26]	BG		x			Human waste at elevated influent feedstock concentration	–
Iordan et al., 2016 [10]	BG				x	Sewage sludge, fats, sludge from septic tanks, and other biological substrates	Analysis for producing 1 MJ of energy
Ke et al., 2022 [28]	BG			x		Cattle manure, swine manure, straw–manure mixture, kitchen waste, and chicken manure	–
Kuo and Dow, 2017 [24]	BG		x		x	fats, oils, and grease (FOG); food waste; and wastewater sludge	45,500 m^3^/day
Laitinen et al., 2016 [30]	BC	x				Wood chips, hog fuel from stumps, bark, sawdust, thermally dried sludge, peat, and SRF (solid recovered fuel)	
Ławniczek-Wałczyk et al., 2012 [31]	BC	x				Agricultural biomass co-combusted with pulverised coal	75 + 200 + 170 MW
Ławniczek-Wałczyk et al., 2017 [32]	BC	x				80% wood chips and 20% agricultural waste (pellets and briquettes, corn briquettes, and sunflower pellet)	205 MW
Madsen et al., 2009 [25]	BC	x				Straw and wood chips	–
Madsen, 2006 [21]	BC	x				Straw and different kinds of wood chips (bark chips with salt water and forest chip).	–
Mbareche et al., 2018 [33]	BG		x		x	Two plants analysed: 1. Primary and secondary sludge from wastewater treatment and organic industrial food waste. 2. Domestic waste under thermophilic conditions.	40,000 + 27,000 tons/year
Merico et al., 2020 [22]	BG	x				Biogas production from agricultural wastes and biomasses	999 kWh_el_ (1069 kWh_t_)
Tolvanen and Hänninen, 2006 [23]	BG		x			Waste treatment through digestion of kitchen biowaste	Volume of reactors: 1600 and 1800 m^3^
Traversi et al., 2015 [27]	BG	x				Agricultural and livestock biomasses	–
Traversi et al., 2018 [29]	BG	x				3 types of plants in relation to the origin of the biomasses introduced into the digester: 3 plants that use ALB (1 in thermophilic and 2 in mesophilic conditions), 1 plant that mainly uses WWTS (mesophilic), and 1 plant that mainly uses OFMSW and FFbP (thermophilic conditions).	3 MW
Zheng et al., 2020 [34]	BG		x			Food waste	300 tons/day

**Table 6:** Overview of the monitoring setup in the analysed plants. FT: Feedstock transport—including operation of handling within the plant facilities before initial treatment; PT: Pre-treatment—initial phases before anaerobic digestion; AD: Anaerobic digestion—sequence of processes by which microorganisms break down biodegradable material in the absence of oxygen; DT: Digestate treatments—operations for processing digestate; DS: Digestate storage—storage of digestate before distribution; CHP: Combined heat and power production through biogas.

Reference	Monitored Phase						Monitoring Method	Sampling Type	Sampling Duration	Feedstocks Employed
	FT	PT	AD	DT	DS	CHP				
Duan et al., 2020 [26]							LCA	–	–	–
Iordan et al., 2016 [10]							LCA	–	–	–
Ke et al., 2022 [28]					x		Analysis on digestate	–	–	–
Kuo and Dow, 2017 [24]			x			x	Portable emission analysers	Portable emission analysers for 1. CH_4_, CO_2_, CO, NO_2_, NO, SO_2_, and O_2_; and 2. formaldehyde, polycyclic aromatic hydrocarbons [PAHs], polychlorinated dibenzodioxins/furans [PCDD/F], and VOCs from combustion fumes	12 weeks	–
Laitinen et al., 2016 [30]	x	x					Environmental and personal monitoring: 1. open face cassettes with polycarbonate filters; 2. adsorption tubes; and 3. real-time measurement	1. Airborne viable bacteria, endotoxins, and fungi; 2. VOCs; 3. Particle dust (PMs)	26′–430′ according to the sample	–
Ławniczek-Wałczyk et al., 2012 [31]	x	x					1. Microbial air sampler; 2. 6-stage Andersen impactor	1. Bacteria and fungi; 2. focus on aerodynamic diameter	1. 1 min and 100 L/min; 2. 5 min and 28.3 L/min	Sunflower seed peel pellets, and wood chips ^1^
Ławniczek-Wałczyk et al., 2017 [32]	x	x					Personal monitoring using conical inhalable samplers	Bacterial pathogens	3 h per 2 times in each location	Wood chips; straw pellets; and corn pellets; sunflower pellets
Madsen et al., 2009 [25]	x						Triplex cyclone + total dust using 25 mm closed-face cassettes. Microorganisms in PM_1_ dust and fungi in total dust were quantified using a modified CAMNEA method	Airborne particles (PM, fungi, and microorganisms)	6 h	Straw and wood chips
Madsen, 2006 [21]	x						CAMNEA filter collection	Airborne microbial component (endotoxins, bacteria, and fungi)	5–7 h per 2 times (including offices)	Bark chips with salt water; straw; forest chips; and industry chips
Mbareche et al., 2018 [33]	x	x	x	x	x		Liquid cyclonic impactor	Fungi and bacteria	9 m^3^ sampled air	Wastewater sludge; food waste
Merico et al., 2020 [22]	Environmental analysis (100 m of a biogas plant)						Ultrasonic anemometer coupled to a thermo-hygrometer (height = 10 m) above the ground, analysers for 1. O_3_; 2. NO, NO_2_, and NO_x_; 3. SO_2_; and 4. CO; PM_2.5_ sampler, an optical particle counter (0.3–20 μm)	VOCs and PM	2 months: 30 January–28 March 2018	–
Tolvanen and Hänninen, 2006 [23]		x	x	x			1. Six-stage impactor and via CAMNEA filter collection; 2. dust sample; 3. noise level.	Concentration of bacteria, fungi, and actinomycetes	1. 30′ 2. 5 L/min for 60–80′	Kitchen waste
Traversi et al., 2015 [27]		x		x			Microbial concentration through air contact on Petri plates, and PM cascade impactor (6 classes: <0.49–10 μm); gravimetric analysis	PM (bioaerosol)	4 h per 12 times (6/plant)	–
Traversi et al., 2018 [29]		x		x			Environmental and personal monitoring: cascade impactor (6 classes: <0.49–10 μm); gravimetric analysis.	PM (bioaerosol)	4 h + 4 h	Agricultural by-products; livestock by-products; food and feed producing by-products; wastewater sludge; and food waste
Zheng et al., 2020 [34]	x	x	x				Portable gas chromatography–mass spectrometer	69 VOCs		–

^1^ With pulverised coal.

**Table 7:** Main detected VOCs identified as being originated from pine and spruce softwood.

	Laitinen et al. (2016) [30]	Zheng et al. (2020) [34]				
Phases of the Process						
Type of Analysed VOCs	Handling of Biomasses at Fuel Reception Hall [μg/m^3^]	Sorting/Crushing Room (SR) [μg/m^3^]	Hydrothermal Hydrolysis Unit (HH) [μg/m^3^]	Anaerobic Digestion Unit (AD) [μg/m^3^]	Biogas Production Unit (BP) [μg/m^3^]	Breakdown of Main Detected VOCs
Terpenes	α-Pinene = 120–5300	1.66 × 10^2^	1.06 × 10^3^	1.5	1.4	Limonene 96%
	Δ3-Carene = 48–3900					
	β-Pinene = 3500					
	Limonene = 23–2200					
	Monoterpene = 2000					
	Campene = 9					
Sulphur-containing compounds	Sulphur dioxide = 45	9.4	44.0	1.5	1.6	Carbon disulphide (45.8%) and dimethyl sulphide (34.6%)
Oxygenated compounds	Acetone = 54	8.74 × 10^3^	3.36 × 10^4^	5.9 × 10^2^	1.68 × 10^2^	Ethanol (91%) and acetone (8%)
Aromatic hydrocarbons	–	7.8	13.4	10.2	8.2	Benzene, toluene, ethylbenzene, and xylene (BTEX–85%)
Halogenated compounds	–	24.9	133.5	20.6	26.7	Ethyl chloride (44%) and dichloromethane (26%)

**Table 8:** Annual average and maximum hourly values in comparison to the limits prescribed by the European Directive 2008/50/CE [35].

Emitted Gases	CO (mg/m^3^)	NO (µg/m^3^)	NO_2_ (µg/m^3^)	NO_X_ (µg/m^3^)	SO_2_ (µg/m^3^)	O_3_ (µg/m^3^)
Annual average	0.29	1.64	10.77	12.60	0.20	66.65
General limit set by the 2008/50/CE (annual)		30	40	30	20	–
Limit value for the protection of human health (annual)	–		26	–	–	–
Hourly maximum	2.76	52.75	70.61	151.21	6.86	114.97
General limit set by the 2008/50/CE (Hourly H, and Daily D)	10 (D)	–	200 (H)	–	350 (H)	180
Limit value for the protection of human health (Hourly H, and Daily D)	5 (D)		100 (H)		50 (D)	
Emitted Particulate						
**Emitted Gases**	**PM_2.5_** **(µg/m^3^)**	**Nanoparticles** **(d < 0.05 µm, n/cm^3^)**	**Ultrafine** **(d < 0.3 µm, n/cm^3^)**	**Accumulation** **(0.3 µm < d < 1 µm, n/cm^3^)**	**Coarse** **(d > 1 µm, n/cm^3^)**	**PM_10_** **(µg/m^3^)**
Average	16.3	6289.9	10,312.9	53.6	0.54	21
Standard Deviation	9.5	7914.7	9620.6	101.4	0.61	10.8
Threshold set by the 2008/50/CE (annual)	25	–	–	–	–	40

**Table 9:** GIMC and MBC indexes. Adapted from [27].

Risk	Indicator	Reference Guideline for Human Health
Microbiological	GIMC	1000 < GIMC < 5000: Intermediate contamination
		GIMC > 10,000: Very high contamination
	MBC (ratio)	MBC < 3: No worsening of the GIMC evaluation
Endotoxin inhalation	EU [EU/m^3^]	90 [56]
		50–200 [57]

## Data Availability

Not applicable.

## Supplementary Materials

The following supporting information can be downloaded at: https://www.mdpi.com/article/10.3390/ijerph20075305/s1, Table S1: Residential setting: Juntarawijit 2013—Results [17]; Table S2: Residential setting: Lee et al. 2021—Results (a) [18]; Table S3: Residential setting: Lee et al. 2021—Results (b) [18]; Table S4: Occupational setting: Schlünssen et al. 2011—Results [19]; Table S5: Occupational setting: Basinas et al. 2012—Results [20].
