# Supplementary material for: Analysing the Impact on Health and Environment from Biogas Production Process and Biomass Combustion: A Scoping Review
Source: Int J Environ Res Public Health. 2023 Mar 29;20(7):5305. doi: 10.3390/ijerph20075305 (PMC10094619; doi:10.3390/ijerph20075305)
Supplement: Supplementary file 1 [file ijerph-20-05305-s001.zip › ijerph-2234151-SI.pdf]

Supplementary materials

Table S1. Residential setting: Juntarawijit 2013 – Results. *Only households; **All members of household. Reference category: > 1.0 km [17].

	**OR (95% CI)**	
	**Residential proximity (km)**	
**Health symptoms (N=392)***	**Exposure group I (0 - 0.5)**	**Exposure group II (0.5 - 1.0)**
Allergic symptoms	2.7 (95% CI: 1.6–4.5)	
Cough	3.9 (95% CI: 2.3-6.6)	
Difficulty breathing	6.7 (95% CI: 3.3–13.6)	3.1 (95% CI: 1.4–6.9)
Eye irritation	5.3 (95% CI: 3.0–9.1)	
Itching/rash	7.2 (95% CI: 4.2–12.5)	
Sore throat	2.5 (95% CI: 1.5–4.4)	
Stuffy nose	8.5 (95% CI: 4.4–16.4)	2.1 (95% CI: 1.0–4.6)°<br>°Borderline significant
**Chronic diseases(N= 1254)****		
Allergy	**2.4** (95% CI: 1.5–4.0)	
Asthma		**2.1** (95% CI: 1.0–4.4)°<br>°Borderline significant
COPD	**2.7** (95% CI: 1.0–8.4)	

Table S2. Residential setting: Lee et al. 2021 – Results a). Reference category: 15 reference sites without biorefineries [18].

	Km	Adjusted RR(95% CI)				
		**All respiratory (N = 547 437)**	**Asthma (N = 507 066)**	**Chronic bronchitis (N = 27 832)**	**Emphysema (N = 1638)**	**Chronic airway obstruction (N = 10 901)**
a) Proximity Biorefinery						
	0–5	3.64 (3.47, 3.81)	3.46 (3.29, 3.64)	4.95 (4.01, 6.13)	18.2 (4.35, 75.9)	5.29 (4.35, 6.42)
	>5–10	1.50 (1.44, 1.56)	1.42 (1.36, 1.49)	3.02 (2.46, 3.71)	n.s.	2.06 (1.71, 2.49)
b) Pollutants						
$PM_{2.5}$ (μg/m^3)						
	0–5	1.15 (1.11, 1.20)	1.10 (1.09, 1.10)	1.13 (1.13, 1.15)	1.25 (1.12, 1.39)	1.14 (1.12, 1.15)
	>5–10	n.s.	1.01 (1.01, 1.01)	1.02 (1.02, 1.03)	n.s.	1.01 (1.01, 1.02)
SO_2 (ppb)						
	0–5	2.07 (1.68, 2.68)	1.59 (1.57, 1.63)	1.83 (1.69, 1.98)	2.98 (1.74, 5.12)	1.87 (1.74, 2.02)
	>5–10	n.s.	1.03 (1.03, 1.04)	1.11 (1.09, 1.13)	n.s.	1.07 (1.05, 1.08)
NO_2 (ppb)						
	0–5	2.17 (1.74, 2.87)	1.64 (1.61, 1.68)	1.89 (1.74, 2.07)	3.19 (1.80, 5.65)	1.95 (1.80, 2.10)
	>5–10	n.s.	1.04 (1.04, 1.04)	1.14 (1.12, 1.17)	n.s.	1.09 (1.07, 1.12)

Adjusted rate ratios (RRs) and 95% confidence intervals of the associations between biorefinery exposures and respiratory diseases according to two exposure indicators: a) residential proximity and b) AERMOD-modelled air pollutant concentrations

Table S3. Residential setting: Lee et al. 2021 – Results b). Reference category: 15 reference sites without biorefineries [18].

	N (%)	Adjusted RR (95% CI)		
**a) Residential proximity (0-10 km)**		**All respiratory (N=276,460)**	**Asthma (N= 256,513)**	**Other respiratory diseases (N= 19,947)**
**Feedstocks**				
All types	276,460 (100)	1.67 (1.65, 1.69)	1.45 (1.45, 1.45)	2.55 (1.08, 2.88)
Corn	1,332 (0.48)	2.59 (2.53, 2.65)	n.s.	4.13 (3.88, 4.39)
Wood	25,050 (9.06)	1.66 (1.64, 1.69)	2.12 (1.98, 2.27)	1.54 (1.51, 1.58)
Soybean	250,078 (90.5)	1.83 (1.79, 1.87)	5.28 (3.95, 7.05)	4.43 (3.35, 5.87)
**b) AERMOD-modelling air pollutants (0-10 km)**				
**Corn**	1,332 (0.48)			
$PM_{2.5}$ (μg/m3)		2.048 (2.013, 2.083)	n.s.	2.906 (2.774, 3.045)
SO_2 (ppb)		1.352 (1.342, 1.362)	n.s.	1.567 (1.536, 1.598)
NO_2 (ppb)		1.922 (1.893, 1.952)	n.s.	2.644 (2.534, 2.759)
**Wood**	25,050 (9.06)			
$PM_{2.5}$ (μg/m3)		1.003 (1.003, 1.003)	1.004 (1.003, 1.004)	1.002 (1.002, 1.002)
SO_2 (ppb)		1.072 (1.070, 1.074)	1.108 (1.098, 1.118)	1.061 (1.058, 1.064
NO_2 (ppb)		1.056 (1.055, 1.058)	1.084 (1.076, 1.092)	1.048 (1.045, 1.050)
**Soybean**	250,078 (90.5)			
$PM_{2.5}$ (μg/m3)		1.377 (1.361, 1.393)	1.377 (1.361, 1.393)	2.197 (2.549, 1.894)
SO_2 (ppb)		1.712 (1.679, 1.746)	1.712 (1.679, 1.746)	3.760 (4.827, 2.928)
NO_2 (ppb)		3.628 (3.462, 3.800)	3.628 (3.462, 3.800)	23.9 (13.1, 43.5)

Adjusted rate ratios (RRs) and 95% confidence intervals of the associations between respiratory diseases and: a) residential proximity (residency within 10 km) to 3 different types of biorefineries (corn, wood and soybean) and b) AERMOD modelled air pollutants in New York State, 2011-2015

Table S4. Occupational setting: Schlünssen et al. 2011 – Results. Reference category: conventional fuel workers [19].

	Biofuel worker group	Exposure level	Significant association with	Adjusted OR (95% CI)
**Endotoxin**	All workers	Most exposed (12.6-294 EU/m^3)	Work-related rhinitis	3.1 (95% CI: 1.1–8.8)
	Straw workers		Asthma symptoms	8.7 (95% CI: 1.1–71.4)
**Total dust**	All workers	Most exposed (0.0.8-0.33 mg/m^3)	Work-related rhinitis	3.2 (95% CI: 1.1–9.2)
			Asthma symptoms	9.4 (95% CI: 1.7–52.0)
**Fungi**	Straw workers	Moderately exposed (3.64 x 10^3 – 8.83 x 10^3 cfu/m^3)	Work-related asthma/wheeze	7.4 (95% CI: 1.1–48.1)
			Asthma symptoms	17.8 (95% CI: 2.3–137)
		Most exposed (9.74 x 10^3 – 1.85 x 10^4 cfu/m^3)	Work-related rhinitis	5.7 (95% CI: 1.4–23.0)
Aspergillus fumigatus	Wood workers	Moderately exposed (224-275 cfu/m^3)	Work-related asthma/wheeze	4.0 (95% CI: 1.6–26.2)
	Straw workers	Moderately exposed (640-1.44 x 10^4 cfu/m^3)	Work-related rhinitis	5.5 (95% CI: 1.2–25.2)
	Straw workers	Most exposed (1.47 x 10^3 -2.78 x 10^3 cfu/m^3)	Work-related rhinitis	4.2 (95% CI: 1.0–18.3)* *Borderline significant

Table S5. Occupational setting: Basinas et al. 2012 – Results [20].

Endotoxin exposure	Significant association with	Adjusted OR (95% CI)
Medium(50-1000 EU/m^3)	Chronic bronchitis	11.05 (95% CI: 1.27–96.35)
High (> 1000 EU/m^3)	Wheezing	5.09 (95% CI: 1.28–20.24)
